# Randomized Controlled Trial of Tailored Music Listening Intervention for Sleep in Dementia

**DOI:** 10.1093/geroni/igaa057.694

**Published:** 2020-12-16

**Authors:** Darina Petrovsky, Shana Roan, Nalaka Gooneratne, Joke Bradt, Laura Gitlin, Nancy Hodgson

**Affiliations:** 1 University of Pennsylvania, Philadelphia, Pennsylvania, United States; 2 Drexel University, Philadelphia, Pennsylvania, United States; 3 University of Pennsylvania, Cherry Hill, New Jersey, United States

## Abstract

Sleep disruption in older adults living with Alzheimer’s disease and related dementias (ADRD) is debilitating and contributes to increased institutionalization, reduced cognitive function, and accelerated disease progression. Given the potential harmful effects of pharmacologic treatment, non-pharmacologic approaches, such as music, may provide a safer alternative to improve sleep quality in this vulnerable population. No empirically validated music protocol exists to address sleep disruption in older adults with ADRD living at home. Therefore, the specific aims of this wait-list randomized controlled trial were to examine the 1) feasibility; 2) acceptability; and 3) preliminary efficacy of a tailored music intervention in home-dwelling older adults with ADRD with sleep disruption and their caregivers. This presentation focuses on baseline characteristics of dyads, which included persons with ADRD and their caregivers who have completed the clinical trial so far (N=28). The mean age of persons with ADRD was 71.6 (SD: 7.6). The mean age of caregivers was 58.7 (SD: 16.7). Sixty-eight percent (n=19) of persons with ADRD were female. Similarly, the majority of caregivers were female (n=20, 71.4%). Seventy-four percent of persons with ADRD scored 0.5 on the Clinical Dementia Rating instrument, indicative of very mild dementia. The majority of dyads identified themselves as non-Hispanic (>92%). Seventy-nine percent of persons with ADRD identified themselves as Black or African-American (n=22, 79%), while 82.1% of caregivers identified themselves as Black or African American. Preliminary analysis of qualitative data indicates high acceptability of the intervention. Results from this research study will inform a future efficacy trial.

